# A review of the diagnosis and management of hyponatremia in connolly hospital: an audit of current practice and the construction of a clinical aid for the diagnosis and treatment of Hyponatremia

**DOI:** 10.1186/1753-6561-9-S7-A16

**Published:** 2015-10-27

**Authors:** Neil McAuliffe, Seamus Sreenan

**Affiliations:** 1 Royal College of Surgeons in Ireland, Dublin, Ireland; 2 Connolly Hospital Blanchardstown, Dublin, Ireland

## Background

Hyponatraemia is the most common example of body fluid and electrolyte imbalance encountered in clinical practice, and is associated with increased mortality, morbidity and length of hospital stay in patients [1]. In spite of this, the diagnosis and management of hyponatremia remains inconsistent as clinicians adopt a broad range of hospital- and specialty-specific approaches [1,2]. In light of this observed inconsistency, the objectives of the present project were: (i) To audit all patients admitted to Connolly Hospital Blanchardstown (CHB) Emergency Department (ED) with hyponatremia (<135 mmol/L) over a 14 day period. (ii) Record the diagnostic and management methods employed, comparing them with recent guidelines published by the European Society of Endocrinology (ESE). (iii) To construct a clinical aid for the diagnosis and treatment of hyponatremia, specific to CHB.

## Methods

The records of all patients admitted to the ED over a 14 day period (N= 426) were studied. Those presenting with hyponatremia (serum sodium <135 mmol/L) upon initial measurement were identified and their lab results and patient files reviewed.

## Results

Hyponatremia (< 135 mmol/L) was observed in 10.7% of admitted patients (n = 46; Sex: 12:34, M:F; Age: Mean : 63.4; Range: [16 – 98]) on initial measurement. Of these, 63% had mild (130-135 mmol/L), 19.6% moderate (125-129) and 17.4% profound (<125) hyponatremia respectively. In 41% of cases (18/44) inappropriate or insufficient diagnostic methods were utilised, when compared with the ESE guidelines. Blood glucose was measured in 69.5% (32/46) of patients, 32.6% (15/46) had Thyroid Function Tests and 17.3% (8/46) had Serum Cortisol measured. In 9% of cases (4/44) the management employed was inconsistent with the guidelines. In addition, 2 incidences (4.5%) of rapid overcorrection of sodium were observed (>10 mmol/L for the first 24 hours and >8 for any 24 hours thereafter).

## Conclusions

Analysis of the data revealed that while the management of hyponatremic patients was largely consistent with ESE guidelines, the diagnostic procedures in many cases were not. These results confirm the need for a diagnostic and management algorithm in CHB, and given the consistency of results across other institutions, the implementation of ESE guidelines in other centres may yield improved patient care and outcomes.
